# TCF7L2 gene polymorphism in populations of f ive Siberian ethnic groups

**DOI:** 10.18699/VJGB-22-23

**Published:** 2022-03

**Authors:** L.E. Tabikhanova, L.P. Osipova, T.V. Churkina, E.N. Voronina, M.L. Filipenko

**Affiliations:** Institute of Cytology and Genetics of the Siberian Branch of the Russian Academy of Sciences, Novosibirsk, Russia Novosibirsk State University, Novosibirsk, Russia; Institute of Cytology and Genetics of the Siberian Branch of the Russian Academy of Sciences, Novosibirsk, Russia Novosibirsk State University, Novosibirsk, Russia; Institute of Cytology and Genetics of the Siberian Branch of the Russian Academy of Sciences, Novosibirsk, Russia Novosibirsk State University, Novosibirsk, Russia; Institute of Chemical Biology and Fundamental Medicine of the Siberian Branch of the Russian Academy of Sciences, Novosibirsk, Russia Novosibirsk State University, Novosibirsk, Russia; Institute of Chemical Biology and Fundamental Medicine of the Siberian Branch of the Russian Academy of Sciences, Novosibirsk, Russia Novosibirsk State University, Novosibirsk, Russia

**Keywords:** Buryats, Teleuts, Yakuts, Dolgans, Russians from East Siberia, type 2 diabetes mellitus, genetic polymorphism, real-time PCR, TCF7L2 (G103894T, rs12255372), TCF7L2 (C53341T, rs7903146), буряты, телеуты, якуты, долганы, русские Восточной Сибири, сахарный диабет 2 типа, генетический полиморфизм, ПЦР в режиме реального времени, TCF7L2 (G103894T, rs12255372), TCF7L2 (C53341T, rs7903146)

## Abstract

Investigation of the frequencies of functionally signif icant gene variants in the context of medical biology and gene geography is a relevant issue for studying the genetic structure of human populations. The transition from a traditional to an urbanized lifestyle leads to a higher incidence of civilizational diseases associated with metabolic disorders, including type 2 diabetes mellitus. The goal of the present paper is to analyze the frequencies of functionally signif icant gene alleles in the metabolic prof iles of indigenous Siberian peoples to identify the gene pool resilience, evaluate the susceptibility of various ethnic groups to metabolic disorders under changing environmental conditions, and predict the epidemiological situation that may occur in the near future. The study was performed in the monoethnic samples of eastern and western Buryats, Teleuts, Dolgans, and two territorial groups of Yakuts. A real-time PCR was used to determine the frequencies of single nucleotide polymorphisms (SNPs) G103894T, rs12255372, and C53341T, rs7903146 in the TCF7L2 gene. The results obtained were compared to the frequencies identif ied for Russians from Eastern Siberia and the values available in the literature. The frequencies of the polymorphic variants studied in the samples from the indigenous Siberian peoples place them in between Caucasian and East Asian populations, following the geographic gradient of polymorphism distribution. A signif icantly lower occurrence of type 2 diabetes risk alleles TCF7L2 (103894T) and TCF7L2 (53341T) in the samples of indigenous Siberian peoples compared to Russians was observed, which agrees with their lower susceptibility to metabolic disorders compared to the newcomer Caucasian population. Taking into account urbanization, a reduced growth in type 2 diabetes incidence may be predicted in indigenous Siberian peoples, i. e. Buryats, Yakuts, Dolgans, and Teleuts, compared to the newcomer Caucasian population. A further study of population structure with respect to different metabolic prof ile genes is required to better understand the molecular genetic foundations of the adaptive potential of indigenous Siberian peoples

## Abstract (ru)

Исследование частот функционально значимых вариантов генов в медико-биологическом и геногеографическом контексте является актуальным направлением в изучении генетической структуры популяций человека. С переходом человечества от традиционного к урбанизированному образу жизни все большее распространение получают болезни цивилизации, связанные с нарушением метаболизма, в том числе сахарный диабет 2-го типа. Цель настоящей работы – проанализировать частоты функционально значимых аллелей генов метаболического профиля у коренных народов Сибири, чтобы определить «запас прочности» генофонда, оценить степень подверженности разных этнических групп заболеваниям метаболического спектра в меняющихся условиях внешней среды и спрогнозировать эпидемиологическую ситуацию в ближайшем будущем. Материалом исследования послужили этнические выборки восточных и западных бурят, телеутов, долган и двух территориальных групп якутов. Методом полимеразной цепной реакции в режиме реального времени определены частоты полиморфных вариантов, обусловленных однонуклеотидными заменами G103894T, rs12255372, и C53341T, rs7903146 гена TCF7L2. Полученные показатели сравнены с частотами в выборке русских Восточной Сибири и с литературными данными. В соответствии с общим географическим градиентом распределения полиморфных вариантов, частоты их в выборках коренных сибирских народов находятся в промежуточном положении между европеоидами и популяциями Восточной Азии. Показана статистически значимо меньшая встречаемость аллелей риска сахарного диабета 2-го типа TCF7L2 (103894T) и TCF7L2 (53341T) в выборках коренных сибирских народов по сравнению с русскими, что согласуется с их меньшей подверженностью метаболическим нарушениям, чем у пришлого европеоидного населения. В условиях урбанизации можно также прогнозировать сниженный рост заболеваемости сахарным диабетом 2-го типа у коренных народов Сибири – бурят, якутов, долган и телеутов, по сравнению с пришлым европеоидным населением. Для более полного понимания молекулярно-генетических основ адаптивного потенциала коренных народностей Сибири необходимо дальнейшее изучение структуры популяций по другим генам метаболического профиля

## Introduction

Investigation into the peculiarities of the population genetic structure of ethnic groups in the context of medical biology and gene geography is a relevant issue in human genetics. To better understand molecular genetic foundations of adaptive potential that ethnic groups develop as they evolve under specific climatic and geographic conditions and adapt to specific dietary patterns, it is important to analyze the frequencies of the candidate gene alleles proven to be functionally significant based on studies in individual populations.

Type 2 diabetes mellitus (DM2) is among the leading mortality and disability factors in a working-age population (Asfandiyarova, 2015). DM2 is a metabolic syndrome component and above that is linked to increased risk of multiple associated pathological states, primarily including cardiovascular diseases (infarctions, strokes, and cardiovascular failure) and chronic renal failure

Incretin hormone secretion defect, a key element of DM2 pathogenesis, is associated with TCF7L2 gene polymorphism since it is this gene’s product that regulates the production of pancreatic β-cells from pluripotent stem cells and is involved in glucose-stimulated insulin secretion (Bennett et al., 2002). In addition, the gene also targets the brain, where TCF7L2 determines the intensity of the anorexigenic effect and affects the central glucose homeostasis mechanism (Ametov et al., 2016). In the liver, the gene is involved in the regulation of triglycerides and low- and very low-density lipoprotein exchange. It is also involved in gluconeogenesis and acts as an insulin resistance mediator (Nobrega, 2013).

It was found that SNPs G103894T, rs12255372, and C53341T, rs7903146 in introns 3 and 4 of gene TCF7L2 were associated with DM2 (Sladek et al., 2007; Timpson et al., 2009; Xi et al., 2014; Katsoulis et al., 2018). The link of TCF7L2 (103894T ) and TCF7L2 (53341T ) alleles with increased risk of DM2 was demonstrated in a number of populations around the world, including Russia (Saxena et al., 2006; Cauchi et al., 2007; Potapov et al., 2010; Bondar’ et al., 2013; Avzaletdinova et al., 2016; Kaya et al., 2017; Melnikova et al., 2020). It was shown that the TCF7L2 (53341T ) variant was linked to increased risk of DM2 compared to TCF7L2 (103894T ), with homozygous alleles showing higher susceptibility to the disease than heterozygous ones (Anjum et al., 2018)

The TCF7L2 polymorphisms are also linked to BMI, total body fat volume, as well as subcutaneous and visceral fat (Haupt et al., 2010; Smetanina, 2015). TCF7L2 (53341T ) allele is associated with the risk of myocardial ischemia and myocardial infarction as syntropic diseases with common pathogenetic elements (Melzer et al., 2006; Han et al., 2010; Orlov et al., 2011). Gene TCF7L2 is also linked to renal embryogenesis, i. e. its polymorphisms are associated with various degrees of chronic renal failure, a vascular complication of DM2 (Franceschini et al., 2012; Ametov et al., 2016; Vikulova et al., 2017). It was proved that TCF7L2 polymorphism in loci rs7903146 and rs12255372 was associated with risks of gastric, breast, and colorectal cancer (Rosales-Reynoso et al., 2016; Zhang et al., 2018). The effect of natural selection on locus rs7903146 in gene TCF7L2 was discovered and a statistically significant link between 53341Т allele frequency and several climatic geographic factors was shown (Trifonova et al., 2020).

Studies on the frequencies of gene alleles associated with the risk of DM2 and other metabolic disorders in indigenous Siberian populations have remained relevant throughout the recent decade (Bairova et al., 2013; Baturin et al., 2017; Hallmark et al., 2018; Kurtanov et al., 2018; Ievleva et al., 2019; Tabikhanova et al., 2019; Melnikova et al., 2020). However, the distribution of the polymorphic variants of functionally significant gene TCF7L2 in Siberian populations remains understudied. Polymorphism frequencies in locus rs7903146 for some Siberian peoples, including Buryats and Yakuts, were presented in (Trifonova et al., 2020). Unfortunately, the authors did not indicate the area where genetic material was collected, which seems necessary for these large heterogeneous ethnic groups populating vast territories

The present paper reports the results of a study into the frequencies of polymorphisms G103894T, rs12255372, and C53341T, rs7903146 in gene TCF7L2 associated with several diseases in the populations of indigenous Siberian ethnic groups, namely Buryats, Teleuts, Yakuts and Dolgans, in comparison to Russians living in Siberia.

## Materials and methods

The genetic material for the present research was collected in the field in 2000–2006. Blood samples were taken from apparently healthy volunteers under their informed consent and with the approval of the local healthcare authorities and the Ethics Committee of the Institute of Cytology and Genetics, SB RAS. Before blood sampling, all volunteers filled in a special demographic questionnaire to specify their ancestors’ nationalities down to 3 to 4 generations.

The data obtained were used to form 7 population samples covering Southern and Eastern Siberia. Persons of Buryat nationality with no outsider ancestors living in Alkhanay and Orlovsky settlements in the Aginsky Buryat Okrug (ABO) of Zabaykalsky Krai were included in the Eastern Buryat group (N = 132). Ethnic Buryats from settlements of Ekhirit- Bulagatsky District of Ust-Ordynsky Buryat Okrug (UOB) of the Irkutsk Region (N = 278) were included in the Western sample. Also included in the study were Teleuts from the Belovo District of the Kemerovo Region (N = 116). Two ethni- cally homogeneous samples of Yakuts were formed as follows: the Nyurbinsky group included the residents of settlements Nyurbachan and Syultsy of the Nyurbinsky District (N = 109), and the Ust-Aldansky group – the residents of the Dyupsya settlement of the Ust-Aldansky District (N = 100). The residents of the town of Dudinka and settlements Volochanka and Ust-Avam of the Taymyr Dolgan-Nenets Okrug of Krasnoyarsk Krai identifying as ethnic Dolgans were included in the Dolgan sample (N = 180). The seventh sample combined Russians from Zabaykalsky Krai and the Irkutsk Region (N = 133).

DNA samples were isolated from the leukocyte fraction of venous blood using the BioSilica kits (Russia). Real-time SNP genotyping in genes TCF7L2 (G103894T, rs12255372) and TCF7L2 (C53341T, rs7903146) was performed applying competing TaqMan-probes complementary to polymorphic DNA segments. Primer and probe designs were selected using the sequences available in the NCBI database (http://www.ncbi.nlm.nih.gov/) with UGENE (version 1.14, http://ugene.unipro.ru/) and Oligo Analyzer (version 1.0.3, https://eu.idtdna.com/pages/tools/oligoanalyzer) software (Table 1).

**Table 1.**
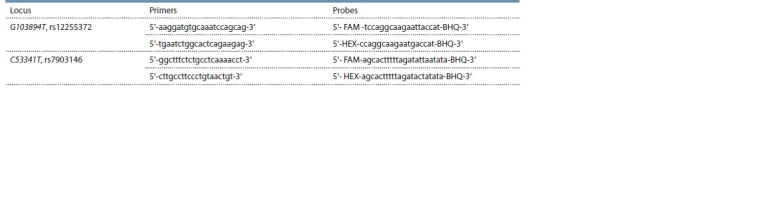
Primer and probe designs used for SNP genotyping in gene TCF7L2

Amplification was performed in 25-μl final volume, the master mix included 300 nM primers, 100 nM TaqMan probes, 65 mM TrisHCl (рН 8.9), 16 mM (NH4)2SO4, 2.5 mM MgCl2, 0.05 % Tween-20, 0.2 mM dNTP, 0.5–10 ng DNA, and 0.5 U Taq DNA polymerase (hot-start, Biosan, IHBFM). Reaction conditions were as follows: initial denaturation for 3 min at 96 °С was followed by 46 cycles including denaturation at 96 °С for 5 s, primer annealing, and extension at 61 °С for 30 s (each step is accompanied by recording fluorescent signals at FAM and HEX fluorophore emission wavelengths).

Allele variant frequencies in the populations were determined based on observed genotype frequencies. The match between empirically observed genotype frequency distribution and theoretically expected distribution at the Hardy–Weinberg equilibrium was tested using Pearson’s chi-squared (the equilibrium holds at p > 0.05). The statistical confidence of allele frequency differences between the studied samples was evaluated using the chi-squared test with Yates continuity correction; the results were considered statistically significant at p < 0.025 (corrected for multiple comparisons, 0.025 = 0.05/2).

## Results

Genotyping results for TCF7L2 (G103894T, rs12255372) and (C53341T, rs7903146) in samples of Buryats, Teleuts, Yakuts, Dolgans, and Russians from Eastern Siberia are presented in Table 2.

**Table 2.**
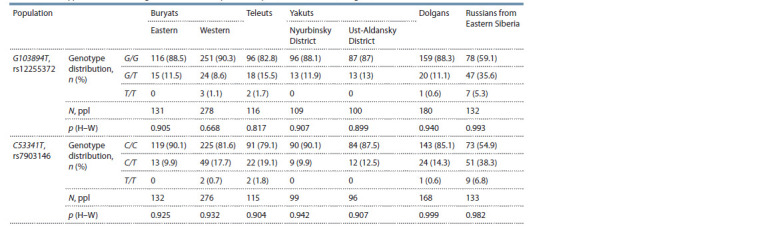
Genotype distribution for gene TCF7L2 in samples of Buryats, Teleuts, Yakuts, Dolgans, and Russians from Eastern Siberia Notе. N is the sample size; n is the quantity; p (H–W) is the probability of Hardy–Weinberg equilibrium deviation.

The genotype distribution matched the Hardy–Weinberg equilibrium for all polymorphic loci and samples. The frequencies of alleles TCF7L2 (103894T ) and TCF7L2 (53341T ) in the studied samples and some ethnic groups described in the literature (The 1000 Genomes…, 2012), as well as comparison of populations ( p-value), are presented in Tables 3–4.

**Table 3.**
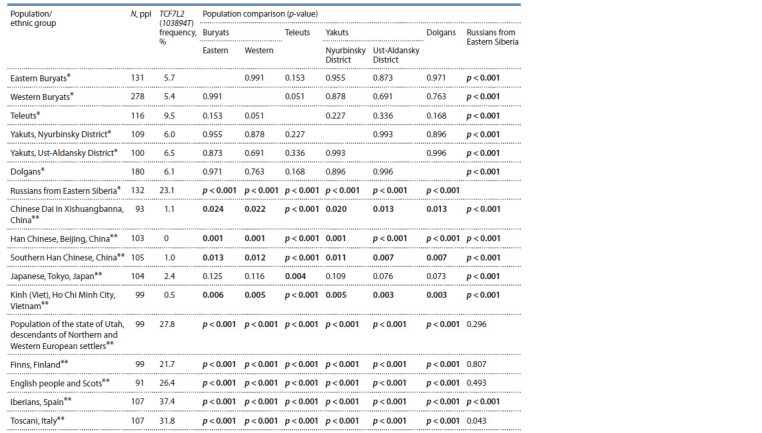
TCF7L2 (103894T) allele frequency in some populations (ethnic groups) and comparison of populations (p-value) Notе. Here and in Table 4: * marks the data obtained by the authors, ** marks the data from the literature (The 1000 Genomes…, 2012); p < 0.025, at which differences were considered statistically significant are marked in bold.

**Table 4.**
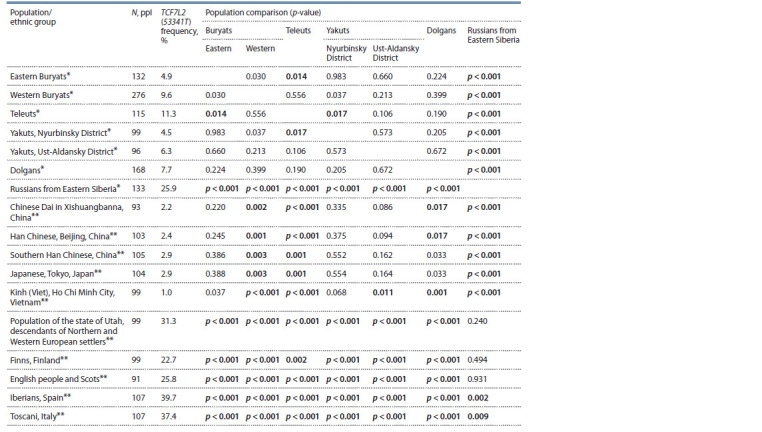
TCF7L2 (53341T ) allele frequency in some populations (ethnic groups) and comparison of populations ( p-value)

It was shown that TCF7L2 (103894T ) allele frequency in the Russians sample (23.1 %) matched that in other Caucasian groups (22–37 %) (The 1000 Genomes…, 2012). The frequency in the studied samples of indigenous Siberian peoples varies from 5.4 % for Western Buryats to 9.5 % for Teleuts, with no statistically significant differences observed. However, the allele frequency in all samples of indigenous populations was significantly lower than in Russians from Eastern Siberia and other Caucasian groups described in the literature (The 1000 Genomes…, 2012). At the same time, it was significantly higher than in several East Asian populations, i. e. Chinese and Vietnamese. We could also see a significant difference between Teleuts and Japanese not observed for other studied groups.

This in-between position of indigenous Siberian populations, as exemplified by Buryats and Teleuts, had been demonstrated earlier in the polymorphism frequencies of some other metabolic profile genes (Tabikhanova et al., 2019).

TCF7L2 (53341T ) allele frequency in the Russians sample (25.9 %) matched that in other Caucasian groups (23–40 %) (The 1000 Genomes…, 2012). The studied samples of indigenous populations showed a significantly lower value compared to Russians varying from 4.5 % in Yakuts from the Nyurbinsky District to 11.3 % in Teleuts. Statistically significant differences were discovered between Teleuts and the samples with the lowest frequency values, namely Eastern Buryats (4.9 %) and Yakuts from the Nyurbinsky District. The data on TCF7L2 (53341T) allele frequency in the samples of Buryats (6.3 %) and Yakuts (4.3 %) resembling the results obtained in our study were presented by Trifonova et al. (2020). Unfortunately, the authors did not indicate sample sizes and the participants’ places of residence, so confidence evaluation was impossible to perform. The frequency values do not show significant differences between the samples of Eastern Buryats and Yakuts from the Nyurbinsky District and the samples of indigenous East Asian populations, namely Chinese, Japanese and Vietnamese, available in the literature. Yakuts from the Ust-Aldansky District demonstrated a significantly higher TCF7L2 (53341T ) allele frequency than Vietnamese, while Dolgans showed differences compared to some Chinese populations as well. Western Buryats and Teleuts showed significantly higher allele frequencies than all the East Asian samples described in the literature. It was also shown that this polymorphism frequency in populations of indigenous Siberian peoples was significantly lower than in the Caucasian groups described in the literature (The 1000 Genomes…, 2012). Thus, TCF7L2 (53341T ) allele frequencies also confirm the trend that places samples of indigenous Siberian peoples in-between East Asian and Caucasian populations

## Discussion

Investigation of the frequencies of functionally significant gene variants in the context of medical biology and gene geography is a relevant issue for studying the population genetic structure of indigenous Siberian peoples. In the present paper, we have determined the frequencies of the 103894T and 53341T alleles in gene TCF7L2 associated with DM2 and other metabolic disorders in the populations of Buryats, Yakuts, Dolgans, and Teleuts, as well as a sample of Russians from Eastern Siberia. It was shown that these frequencies in Russians fall within the same range as in other Caucasian populations. Meanwhile, the populations of indigenous Siberian ethnic groups show significantly lower TCF7L2 (103894T ) and TCF7L2 (53341T ) polymorphism frequencies, which places them in-between Caucasian and East Asian populations.

It was shown in several papers that indigenous Siberian and Far Eastern ethnic groups, as well as the ethnic groups from the European part of Russia with a mongoloid component in their gene pool, had lower incidence rates of metabolic syndrome and its DM2 component compared to Caucasians (Tsyretorova et al., 2015; Kichigin et al., 2017; Cygankova et al., 2018). It is primarily explained by the traditional lifestyle implying a sufficient amount of physical activity and diet consisting mostly of animal source foods rich in proteins and fats with limited carbohydrate component (Bairova et al., 2013).

Ethnic peculiarities in DM2 prevalence and manifestations are also caused by distinctions from the European gene pool, i. e. a unique combination of frequencies of functionally significant genes developed as a result of adaptation to local environmental conditions (Baturin et al., 2017; Hallmark et al., 2018). Differences in living conditions between indigenous and newcomer populations are alleviated due to urbanization, centuries-long traditions, and acquired dietary patterns change, and, as a result, civilizational diseases associated with metabolic disorders, including DM2, become increasingly common in indigenous populations (Ovsyannikova et al., 2007; Tsyretorova et al., 2015; Cygankova et al., 2018). Investigation of the polymorphism distribution of the functionally significant genes associated with risks of diseases in indigenous Siberian populations makes it possible to identify the gene pool resilience, evaluate the susceptibility of various ethnic groups to metabolic disorders under changing environmental conditions, and predict the epidemiological situation in the near future.

Lower prevalence of DM2 among indigenous Siberian populations agrees with reduced populational frequencies of studied alleles TCF7L2 (103894T ) and TCF7L2 (53341T ) associated with DM2 and several syntropic diseases, discovered in the present paper. The reduced frequencies of these polymorphisms may affect the incidence rates of the diseases in the studied populations. With urbanization taken into account, one might predict reduced growth in incidence rates of DM2 and other pathological states associated with studied polymorphisms in indigenous Siberian ethnic groups compared to newcomer Caucasians.

The high frequency of TCF7L2 (53341T ) polymorphism in Teleuts from the Kemerovo Region compared to Buryats and Yakuts may be attributed to a Caucasian component that this ethnic group adopted in their gene pool in the process of formation (Ostaptseva et al., 2006). With more comfortable living conditions close to cities of Prokopyevsk, Kemerovo, and Novokuznetsk and a richer European-type diet, Teleuts may face an increased risk of DM2 and associated diseases. Increased incidence of cardiovascular diseases has been observed in this ethnic group in recent decades (Ovsyannikova et al., 2007). However, polymorphism frequencies of other functionally significant genes are to be investigated to draw better-grounded conclusions.

## Conclusion

Ethnic peculiarities in the frequency distribution of polymorphisms in gene TCF7L2 (G103894T, rs12255372) and (C53341T, rs7903146) in the populations of Buryats, Yakuts, Dolgans, and Teleuts, as well as a sample of Russians from Eastern Siberia, have been studied in the present paper. Locus rs12255372 has been studied in various territorial groups of Buryats and Yakuts for the first time, and the same goes for loci rs12255372 and rs7903146 in the Dolgan and Teleut populations. It has been shown that the samples of indigenous Siberian populations fall in-between Caucasian and East Asian populations with respect to studied polymorphism frequencies, following the geographic polymorphism distribution gradient.

Significantly lower occurrence of TCF7L2 (103894T ) and TCF7L2 (53341T ) alleles associated with DM2 and other metabolic disorders in the samples of indigenous Siberian peoples compared to Russians was demonstrated, which agrees with their lower susceptibility to metabolic disorders, including DM2, compared to the newcomer Caucasian population described in the literature. With the transition to urbanized lifestyle taken into account, one might predict reduced growth in incidence rates of DM2 and other pathological states associated with the studied polymorphisms in indigenous Siberian ethnic groups, namely Buryats, Yakuts, Dolgans, and Teleuts, compared to newcomer Caucasians.

To better understand the nature of ethnic differences, further investigation into population structure with respect to other metabolic profile genes is required.

## Conflict of interest

The authors declare no conflict of interest.
